# Case Report: Identification of novel *CDH2* mutation (p. P183A het)-induced arrhythmogenic cardiomyopathy in China

**DOI:** 10.3389/fcvm.2023.1258807

**Published:** 2023-11-21

**Authors:** Kun Li, Yifei Wang, Jing Yang, Fang Liu, Fulan Liu, Ping Zhang

**Affiliations:** Department of Cardiology, School of Clinical Medicine, Beijing Tsinghua Changgung Hospital, Tsinghua University, Beijing, China

**Keywords:** ACM, *CDH2*, cadherin-2, mutation, adolescence

## Abstract

**Background:**

Arrhythmogenic cardiomyopathy/dysplasia (ACM) is an inheritable heart disease closely related to gene variations induced heart fibrofatty replacement, which increases the risk of arrhythmia events and even sudden cardiac death. In this study, we reported a 10-year-old patient with a novel mutation diagnosed with ACM.

**Case presentation:**

We present the case of a 10-year-old patient admitted with recurrent palpitation, whose electrocardiogram suggested the existence of right ventricle origin premature ventricular contractions and ε wave. Furthermore, echocardiography showed an enlarged right ventricle corrected to a body surface area of 29.57 mm/m^2^. The diagnosis of ACM was clear. Further gene sequencing revealed a novel heterozygous missense mutation of *CDH2* (cadherin-2) c.547C > G (p. P183A) that potentially increases ACM risk by affecting adherens junctions of the intercalated discs.

**Conclusions:**

This is the first case of *CDH2* mutation (c.547C > G, p. P183A) related ACM in the Chinese population. Compared to previously reported mutations inducing ACM by affecting desmosome function, the newly reported *CDH2* variation revealed a novel potential mechanism that induces ACM by disturbing cell-cell adhesion.

## Introduction

Arrhythmogenic cardiomyopathy/dysplasia (ACM) is an inheritable heart-muscle disorder that is characterized by fibrofatty replacement of heart ventricular myocytes, pre-dominantly, the right ventricular (RV), which increases the risk of potential ventricular arrhythmia and sudden cardiac death (SCD). The prevalence of ACM is approximately 1:2,500 (1). ACM is a major inducement of SCD in young people and athletes, especially for patients <30 years of age (2).

At present, the diagnosis of ACM mainly relies on the updated Task Force criteria released in 2010 for adults, which is not sensitive for children and patients at an early stage. A positive genetic test result can help confirm the diagnosis and further be used in a risk prediction model (3). Previous studies about the molecular mechanisms of ACM suggested that the generation and development are closely related to mutations of genes encoding desmosome-related proteins, which are involved in ventricular structure maintenance and signal communication. These genes include plakoglobin (*JUP*), desmoplakin (*DSP*), plakophilin2 (*PKP2*), desmocollin2 (*DSC2*), desmoglein 2 (*DSG2*), and desmin (*DES*). Furthermore, some genes that encode proteins not related to desmosomes are also reported to be pathogenic, and a total of 15 genes have been identified to be associated with ACM so far (4). Specifically, in 2,017, Mayosi et al. (5) and Turkowski et al. (6) respectively reported that *CDH2*, which encodes cadherin-2, a protein that functions in cell adhesion, was associated with ACM. To our knowledge, no *CDH2* mutation-induced ACM case in China has been reported since then.

In this study, whole-exome sequencing (WES) was applied to explore the candidate gene in a patient with suspected ACM. Following clinical investigation and WES, *CDH2* was confirmed as a novel ACM gene for the first time in China.

## Case report

**Figure 1:**
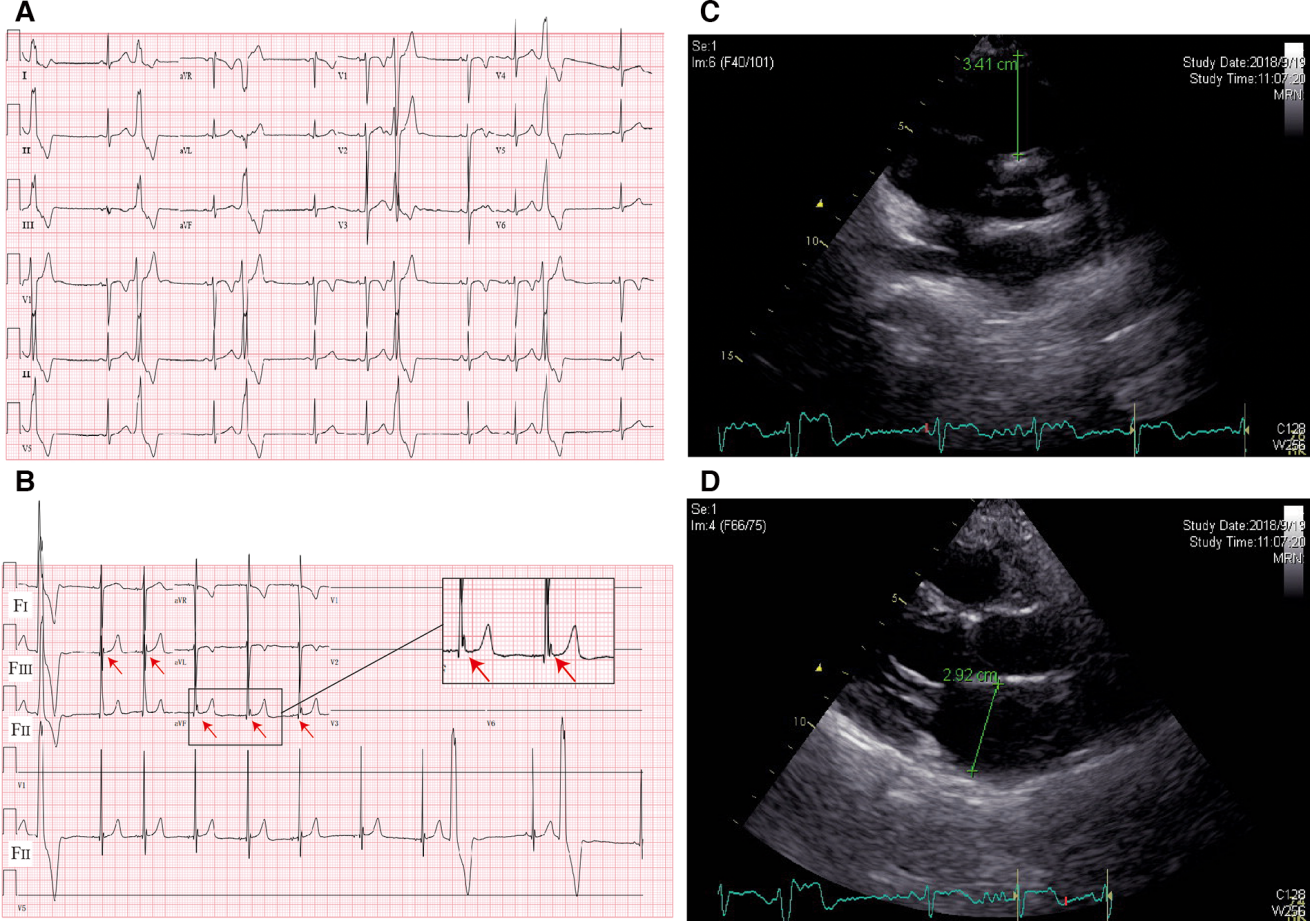
ECG and imaging characteristics of the proband. (**A**) Standard 12-lead ECG; (**B**) fontaine-lead ECG. Note the *ε* wave (arrows) observed only with F-ECG in F_III_ and aVF. (**C**) Echocardiography indicated RVOT enlargement; (**D**) echocardiography indicated LA enlargement.

**Figure 2:**
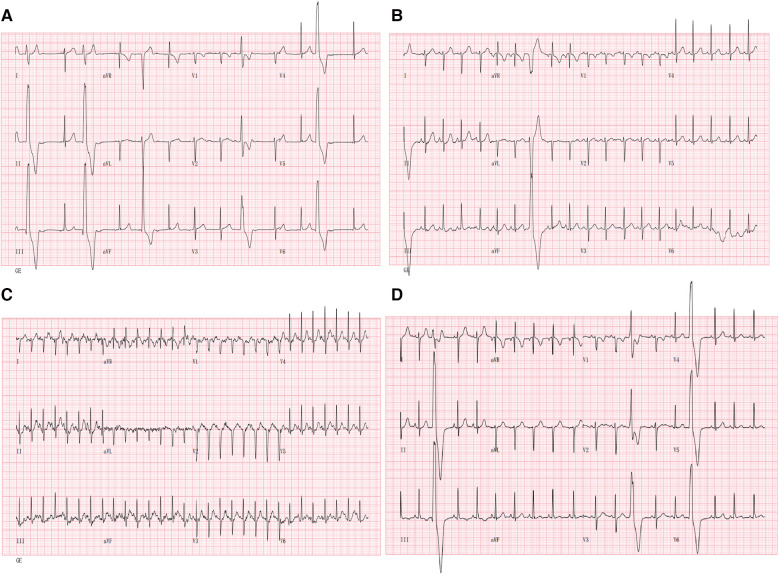
Suppression with exercise: 12-lead ECG during treadmill exercise testing is shown. (**A**) 12-lead ECG at the beginning of exercise; (**B**) 12-lead ECG during exercise (05:50); (**C**) 12-lead ECG during exercise (at peak exercise after 10 min). (**D**) 12-lead ECG during the recovery phase (02:30) of exercise. Note the suppression of PVCs during exercise.

A 10-year-old boy was admitted to our department because of palpitation for 2 years. An initial assessment of the cardiovascular system was performed through an electrocardiogram (ECG), which showed premature ventricular contractions (PVCs) with a wave morphology consistent with a right ventricle (RV) origin and T wave inversion in V1 (Figure 1A). A Fontaine lead ECG(F-ECG) indicated ε wave F_III_ and aVF (Figure 1B). The 24-h Holter indicated frequent PVCs and the total ventricular premature beat count was 18,115 (14%). Moreover, echocardiography demonstrated an enlarged right ventricle (RVOT 34 mm) and left atrial (LA 29 mm) (Figures 1C,D). The body surface area (BSA) of the patient was 1.15 m^2^, and the corrected RVOT/BSA = 29.57 mm/m^2^. Subsequent treadmill exercise electrocardiography tests under the Bruce protocol were done and revealed exercise-induced PVCs of RVOT origin and the PVCs were suppressed at higher heart rates (Figure 2). After investigation, other special diseases including viral infection and coronary artery disease were excluded.

In this case, the diagnosis of ACM was made according to modified Task Force criteria, and the patient met two major and one minor criteria as stated above. To further confirm the diagnosis, peripheral blood lymphocytes from the patient and his family members were collected for genomic DNA extraction. WES was applied to search for candidate gene mutations. The study participant gave informed consent and the study protocol was approved by the Beijing Tshinghua Changgung Hospital IRB.

**Figure 3:**
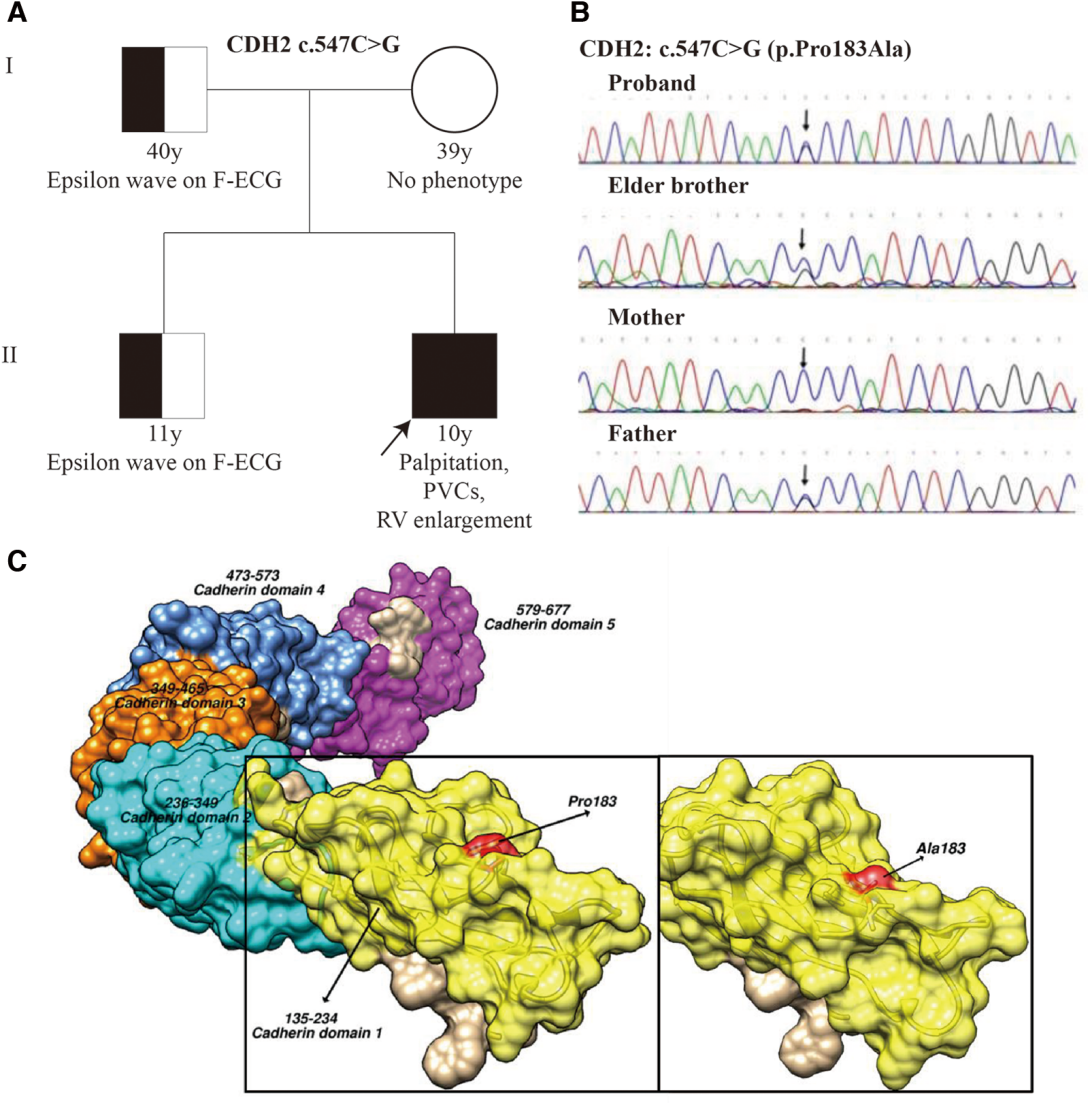
Pedigree and *CDH2* mutation analysis. (**A**) The pedigree of the patient. Squares indicated male family members and circles denoted female family members. (**B**) Sanger sequencing shows a heterozygous missense mutation, c.547C > G (p. P183A), in exon 5 of the CDH2 gene of the proband for the elder brother and father. The arrow indicates the mutation site. (**C**) The predicted 3D structure of *CDH2* protein with wild-type A and mutated type B(pGenTHREADER). The yellow color indicates the functional region located by the mutated amino acid (183Pro-Ala) which is highlighted by the red color.

The genetic test indicated that the patient carried a heterozygous missense mutation of *CDH2* c.547C > G (p. P183A), which was detected by WES and confirmed by subsequent Sanger sequencing (Figures 3A,B). The population frequency of the *CDH2* c.547C > G (p. P183A) mutation was not recorded in ESP6500, 1,000 Genomes, or our local databases while ExAC showed a very low frequency (8.245e-06). The “proline” in the No.183 amino acid of *CDH2* protein is reported to be highly conserved among vertebrates. The missense mutation in the No.547 nucleotide led to the replacement of its coded amino acid nonpolar proline by nonpolar alanine, which was predicted to be probably damaging, deleterious, and disease-causing respectively by three programs for analyzing protein functions, polyphen2, SIFT, and MutationTaste. The *CDH2* c.547C > G (p. P183A het) mutation did not change the length of the mutation-located functional region and the 3-dimension structure of its around residues (Figure 3C). Nevertheless, the No. 183 amino acid connected with the nearby residues via hydrogen bonds as well as σ bonds to oxygen atoms and the 183Pro-Ala mutation could damage the σ bonds and other bonds instead of hydrogen bonds, which would be detrimental to the stability of the functional region. This variant was also verified in his father and elder brother who were asymptomatic (Figures 3A,B) but *ε* waves were confirmed on their F-ECGs.

A low-dose of sotalol was prescribed and he was provided an exercise prescription and advised to refrain from intense or endurance exercise. He still has recurrent palpitations and frequent PVCs after a follow-up of 1 year and refuses further treatment for personal reasons.

## Discussion

In this report, we presented the case of a 10-year-old boy with *CDH2* gene mutation-related ACM, which led to palpitation, frequent PVCs, and RV enlargement. To the extent of our knowledge, this report is the first to describe this recently identified *CDH2*-related ACM in a Chinese patient. In the two previously reported cases (a European family and a white South African family), described by Turkowski et al. and Mayosi et al. respectively (5, 6), the patients were mainly children and adolescents, with more severe clinical presentation including exercise-induced syncopal events, frequent PVCs mainly originating from the RVOT, dilated RV with reduced function, and even SCD. The relatively mild and atypical symptoms make the diagnosis of our patient a challenge.

Clinically, three phases have been described in ACM disease progression including the concealed phase, overt electrical phase, and diffuse and progressive phase (7). Since ACM becomes clinically apparent after 20–40 years (8, 9), it is an easily missed diagnosis and misdiagnosis in the early stage. Cardiac magnetic resonance (CMR) could add incremental value to the diagnosis of cardiomyopathy owing to its ability to assess many different tissue properties. However, CMR is more valuable in the progressive phase of the disease when patients exhibit structural and functional abnormalities. Although our patients are in the early stages of the disease, close follow-up is still necessary to observe changes in cardiac structure and function, particularly paying attention to the presence of myocardial fibrosis, which is a significant risk factor for SCD. As supplementary, a screened pathogenic mutation is regarded as a major criterion and contributes up to 50% of the diagnosis of ACM, especially for the concealed phase and overt electrical phase patients (3). In our present report, we detected a variant of *CDH2* p.Pro183Ala het by WES, which helped us confirm the diagnosis of ACM in a child with atypical clinical manifestation and his asymptomatic family members. The variant is a heterozygous missense mutation in exon 5 and has not been reported before. Therefore, it is necessary to expand the scope of the family investigation. On the one hand, this can help us determine the genetic pattern of the disease and promote research into the disease mechanism. On the other hand, it can lead to the early detection of asymptomatic patients, allowing for regular follow-ups to prevent SCD.

The human *CDH2* gene spanned approximately 225 kb and encoded Cadherin-2, a transmembrane protein mainly involved in cell-cell adhesion in multiple tissues (10). Specifically, cadherin-2 was expressed in cardiac muscle as an integral component in adherens junctions of the intercalated discs, functioning to couple adjacent cardiomyocytes mechanically and electrically. Cadherin-2 is a protein that is 875 amino acids in length, comprising 5 extracellular cadherin repeats (EC1–EC5), a transmembrane region, and a highly conserved cytoplasmic tail that links cadherins to the cytoskeleton, in many cases via sequential binding of b-catenin to a-catenin and then to actin (11). The mutation (p.Pro183Ala) identified in the patient was located in the EC1 region, which is crucial for the adhesive function of cadherin-2. Since the repeats were important for Ca^2+^ binding for correct protein folding (5, 6, 12), the mutation highly likely affected the adhesive function of cadherin-2. In animal models, the deletion of cadherin-2 was proved to disturb the stability of intercalated disc structure and induce the loss of desmosome and adherens function. The mice after cadherin-2 deletion also presented spontaneous ventricular arrhythmias and eventually SCD (12).

To clarify whether the *CDH2* p.Pro183Ala het mutation could influence the function of Cadherin-2, we performed in silico analysis and polyphen2, SIFT, and MutationTaste predicted p.Pro183Ala mutation to be probably damaging, deleterious, and disease-causing respectively. Furthermore, we also confirmed the *CDH2* protein 3D structure and analyzed its conformation changes. The *CDH2* protein usually forms dimer and polymer and extracellular (EC) peptide chains folded to cadherin repeats with Ca^2+^ combined inside. The dimer was stabilized by the cadherin domain 1(CD1) β folding and cadherin domain 2(CD2) Loop linked via hydrogen bond. In our study, we found that the *CDH2* mutation (p. P183A het) was located in the β folding convex and therefore it could damage the stability of the dimer structure (Figure 3C). Further functional study should be done to clarify the role of *CDH2* in ACM.

Therefore, although ACM has been traditionally considered a desmosomal disease, more evidence has appeared to prove that novel mechanisms involving area composits may have a function in the pathogenesis of ACM (3). Unlike desmosomes anchored to the IFs of the cytoskeleton, cadherin-2 is primarily anchored to the actin microfilaments of the cytoskeleton and promotes cell–cell adhesion and communication (3, 4). With the availability of WES, the number of pathogenic genes that can be studied in a single patient rapidly increases. Therefore, a list of core genes focused on those with sufficient disease-related evidence is not enough to account for the disease mechanism. Herein, we report a case of ACM associated with a novel mutation (p. P183A) of the *CDH2* gene, supporting the viewpoint that “non-desmosomal” genes such as *CDH2* may underlie a cardiomyopathy with increased arrhythmic propensity in humans.

Furthermore, while ACM patients are normally asymptomatic early in the disease process, they may present clinically with palpitations, syncope, or even SCD due to ventricular arrhythmias, especially with exertion (13). SCD may be the first clinical manifestation and was reported to account for 20% of deaths in young people and athletes caused by previously undiagnosed ACM (14), which makes the ACM diagnosis in its early stage more important. Despite *CDH2* mutations not being a frequent cause of ACM, a candidate-gene analysis screening of the *CDH2* gene is of vital significance, because *CDH2* mutations may explain a proportion of the 40% of genotype-negative patients with ACM (5).

## Conclusions

Our present manuscript describes a challenging case of ACM with a very rare cause of damage to the stability of cell–cell adhesion, which is a novel mutation (p. P183A) of the *CDH2* gene that plays a critical role in heart development and function and maintaining the structural integrity of the heart. Although according to the HRS guideline for ACM released in 2019, *CDH2* was not included in the minimum set of 15 genes recommended for the detection of arrhythmogenic cardiomyopathy, our study increases the awareness of cadherin-2 as a novel pathogenetic basis for ACM in humans and contributes to genetic counseling of families with ACM, especially for young patients in the early stage.

## Data Availability

The datasets presented in this study can be found in online repositories. BioSample accession number: SAMN36425902.
